# Effect of metal dopants on the electrochromic performance of hydrothermally-prepared tungsten oxide materials†

**DOI:** 10.1039/d3ra06018g

**Published:** 2023-12-19

**Authors:** Kunyapat Thummavichai, Thi Hai Quyen Nguyen, Giulia Longo, Dayuan Qiang, Guillaume Zoppi, Derck Schlettwein, Pietro Maiello, Nicole Fleck, Nannan Wang, Yanqiu Zhu

**Affiliations:** a Department of Mathematics, Physics and Electrical Engineering, Northumbria University NE1 8ST Newcastle UK kunyapat.thummavichai@northumbria.ac.uk; b Institute of Applied Physics, Center for Materials Research (ZfM/LaMa), Justus-Liebig University 35392 Giessen Germany; c School of Mechanical Engineering Sciences, University of Surrey GU2 7XH Surrey UK; d State Key Laboratory of Featured Metal Materials and Life-cycle Safety for Composite Structures, School of Resources, Environment and Materials, Guangxi University 530004 Nanning China; e College of Engineering, Mathematics and Physical Sciences, University of Exeter EX4 4QF Exeter UK

## Abstract

Electrochromic (EC) glass has the potential to significantly improve energy efficiency in buildings by controlling the amount of light and heat that the building exchanges with its exterior. However, the development of EC materials is still hindered by key challenges such as slow switching time, low coloration efficiency, short cycling lifetime, and material degradation. Metal doping is a promising technique to enhance the performance of metal oxide-based EC materials, where adding a small amount of metal into the host material can lead to lattice distortion, a variation of oxygen vacancies, and a shorter ion transfer path during the insertion and de-insertion process. In this study, we investigated the effects of niobium, gadolinium, and erbium doping on tungsten oxide using a single-step solvothermal technique. Our results demonstrate that both insertion and de-insertion current density of a doped sample can be significantly enhanced by metal elements, with an improvement of about 5, 4 and 3.5 times for niobium, gadolinium and erbium doped tungsten oxide, respectively compared to a pure tungsten oxide sample. Moreover, the colouration efficiency increased by 16, 9 and 24% when doping with niobium, gadolinium and erbium, respectively. These findings suggest that metal doping is a promising technique for improving the performance of EC materials and can pave the way for the development of more efficient EC glass for building applications.

## Abstract 2

We investigated the effects of niobium, gadolinium and erbium doping on tungsten oxide using a single-step solvothermal technique. The findings suggest that metal doping can significantly advance electrochromic glass technology, potentially transforming energy efficiency in buildings.

## Graphical abstract

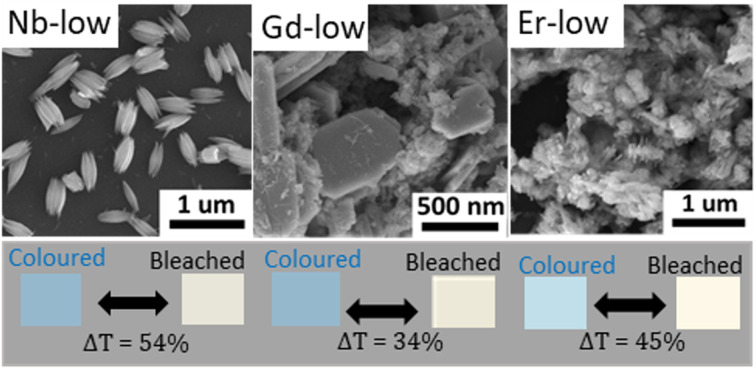

## Introduction

Windows are a fundamental aspect of modern building design and can contribute up to 25% of heat loss in buildings in the winter and up to 40% of unwanted heat gain in the summer.^1^ Smart glass is a promising solution, as it has the capability to optimize the energy efficiency of buildings by regulating the transmission of heat and light through windows, resulting in optimized energy usage and improved indoor comfort for occupants.^2^ Among various types of smart glass, electrochromic (EC) glass has gained more research interest due to its ideal outdoor readability and ability to control the level of transparency in manual mode, making it more suitable for building envelopes compared to other types of smart glass. In addition, EC glass offers the ability to maintain its optical state for a period of time without requiring continuous electrical power, making it an ideal candidate for super energy-saving applications^3^ with up to 20% building energy efficiency improvements compared to normal glass.^4^

Typically, EC devices are composed of multiple layers, including a working electrode layer, ion conductive layer, and ion storage layer.^5^ The underlying process of EC is widely accepted to be a result of insertion/de-insertion of electrons and cations (*e.g.* Li^+^, Na^+^ or H^+^) in the oxide matrix. The insertion reaction into the oxide film can cause a reduction of EC materials' oxidation state, translating into a modification of the electronic structure of the material. As a consequence, the visible photon absorption is modified, leading to a perceivable colour change.^6^

Different type of materials such as conjugated conducting polymers,^7^ transition metal oxides^8,9^ as well as metal coordination complexes^10^ have been intensively studied as EC materials for several decades. Depending on the specific requirements of a given application, the performance of EC devices can be optimized through careful selection of either organic or inorganic EC materials. Both organic and inorganic EC materials offer unique advantages depending on the application at hand, but transition metal oxides are the preferred inorganic EC materials for smart window applications due to their superior properties *i.e.* high correlation coefficient, high stability *etc.* Among those, tungsten oxide (WO_*x*_) is particularly attractive due to its ability to provide high transmittance modulation over a wide temperature range.^11^ However, there are still significant challenges to be addressed such as slow switching times, low coloration efficiency, and degradation issues.^12^

Doping the host materials with metal ions with various oxidizing capacity could serve as the crucial factor in enhancing colour efficiency, durability, and switching time of EC devices. By using this approach, the EC properties of the material can be optimized, resulting in improved device performance and increased efficiency. Various transition metals such as molybdenum (Mo), titanium (Ti), aluminium (Al), vanadium (V), nickel (Ni), and niobium (Nb)^13–18^ have been extensively studied for their electrochromic (EC) performance. On the other hand, rare earth metal elements such as dysprosium (Dy), lanthanum (La), yttrium (Y), erbium (Er)^19–21^ have proven their potential as dopants for enhancing photocatalytic activity. However, only a few of these, such as cerium (Ce) and gadolinium (Gd) have been the focus of studies on overall EC performance.^22,23^

In this study, we explored the utilization of alternative metal elements, specifically Nb, Gd, and Er, to effectively modify the properties of WO_*x*_, aiming to achieve enhanced EC performance. Our work aims to contribute to the development of more efficient EC devices by advancing our understanding of the effects of metal doping on WO_*x*_ materials.

## Experimental details

### Material and chemical

Tungsten hexachloride (WCl_6_), niobium(v) chloride, anhydrous, powder, 99.995% trace metals basis (NbCl_5_·7H_2_O), gadolinium(iii) chloride hydrate 99.99% trace metals basis (GdCl_3_·6H_2_O), erbium(iii) chloride hexahydrate, 99.9% trace metals basis (ErCl_3_·6H_2_O), cyclohexanol, and acetonitrile (ACN) were purchased from Merck and used without any further treatment. Lithium perchloride (LiClO_4_, 99.99%) and propylene carbonate (PC) were purchased from Sigma-Aldrich.

### Metal ions doped tungsten oxide nanostructures preparation

Pure WO_*x*_, Nb, Gd and Er doped WO_*x*_ powder samples are prepared using a one-step solvothermal method. WCl_6_ and cyclohexanol are used as precursor and organic solvent, respectively. For the pure WO_*x*_ sample, WCl_6_ (0.099 g) is dissolved into 50 ml of cyclohexanol to form a solution of 0.005 M. For the doped samples, a 1 ml pre-solution is obtained by dissolving NbCl_5_·7H_2_O into water, while GdCl_3_·6H_2_O and ErCl_3_·6H_2_O are dissolved into ethanol (selected based on the solubility of the precursor), accordingly to the desire molar ratio. This pre-solution is then added into the 49 ml WCl_6_ solution and then mixed under ultra-sonication for 30 min. This homogeneous solution is then transferred into a 120 ml Teflon-lined stainless-steel autoclave and heated up at 200 °C for 10 h. The molar ratio of dopants and WCl_6_ investigated are 1 : 4, 1 : 8 and 1 : 16 for reach of the 3 dopants. After reaction, the autoclave is cooled down naturally in the oven. All the obtained samples are then washed with distilled water, ethanol, and acetone three times and then centrifugally collected (using 8000 rpm for 10 min). They are naturally dried at room temperature in a fume hood for a few days and are collected for further characterisation and testing. To facilitate the reading of this work, the molar ratios of 1 : 4, 1 : 8, and 1 : 16 of dopant : W are referred as “high”, “mid” and “low”, respectively. As example, Nb-high indicates the Nb : W = 1 : 4 sample, while Er-low represents the Er : W = 1 : 16 sample.

### Thin film preparation

The thin films are prepared using a suspension of 0.04 g of the previously described doped-WO_*x*_ in 2 ml ACN solvent, ultrasonicated for 1 h. This well-mixed suspension is spincoated onto an ITO glass substrate (10–12 Ω per sq., 33 mm × 25 mm × 1 mm) at 3000 rpm for 30 s. This process is repeated 10 times to achieve the desired thickness. After that, the thin film is dried in the oven at 80 °C for several hours. The thin film thickness is confirmed by stylus profilometry.

### Samples characterisation

The crystallinity of WO_*x*_-based thin film coated on ITO glass is analysed using X-ray diffraction (XRD, Rigaku SmartLab SE) with a CuKα radiation source (*λ* = 0.15406 nm) operating at 2 kW equipped with Ni CuK filter. Morphologies of all samples are identified by scanning electron microscopy (SEM, Tescan Mira 3) at an operating voltage of 6 kV. The surface chemical composition of the materials is characterised by X-ray photoelectron spectroscopy (XPS, Thermo Scientific K-Alpha), with a 180° double focussing hemispherical analyser. The X-ray source employed is monochromatic Al Kα. Due to the insulating nature of the samples, a combination of low-energy electrons (with energy less than 5 eV) and positively charged argon ions (Ar^+^) is used to prevent charge accumulation. The X-ray spot size employed for all analyses is set at a radius of 400 μm. The Casa XPS analysis program is used for all XPS spectra with subtraction of a Shirley background and Gaussian/Lorentzian (GL) line shape. The EC performance of the samples is evaluated *via in situ* three electrodes system spectroelectrochemical analysis which performs the electrochemical and optical characterizations simultaneously during the operative cycle. The EC thin film, platinum wire (Goodfellow, 99.995%), and Ag/AgCl leak-free (Innovative Instruments, Inc.) are used as working, counter and reference electrodes, respectively. 0.94 M LiClO_4_/PC is used as electrolytes. Cyclic voltammetry (CV) is carried out by a potentiostat/galvanostat (Ivium Technologies B.V.), scanning between −1.2 and 1.2 V at different scan rates of 20, 40, 60, 80 and 100 mV s^−1^. Chronoamperometry (CA) is recorded on the optimal samples, switching between −1.2 and 1.2 V at a time interval of 60 s. To analyse the intercalation/de-intercalation of the Li^+^ ions within the films, −1.2 V bias potential is applied for different times, from 1 to 300 s. The optical spectra are simultaneously recorded with a UV-Vis spectrometer (Tec-5, Steinbach).

The effective diffusion coefficient of Li^+^ (*D*_Li^+^_ in cm^2^ s^−1^) is calculated using Randles–Ševčík's equation (eqn (1)),^22^ by assuming a simple diffusion-controlled process.1
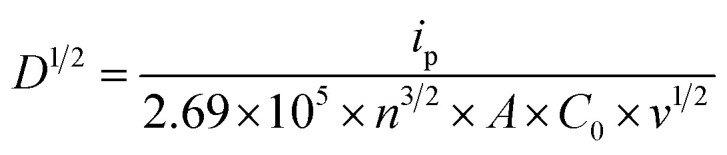
where *i*_p_ is the peak current, *n* is the number of electrons transferred in the redox reaction (*n* = 1 in this case), *A* is the surface area of the working electrode (in cm^2^), *C*_0_ is the bulk concentration of the electrolyte (0.94 mol cm^−3^), and *ν* is the scan rate (V s^−1^). The transmittance change of all samples is calculated based on eqn (2) at a specific wavelength of 710 nm.^24^

2 Δ*T* = (*T*_bleached_ − *T*_coloured_)_*λ*=710_

The colouration efficiency (CE) of all sample is calculated from the change in optical contrast density (ΔOD) per the total charge that passes across the unit area of the thin film electrode (*Q*_d_, C cm^−2^) as shown in eqn (3) and (4).^25^

3 CE = ΔOD/*Q*_d_

where

4 ΔOD(*λ*) = log(*T*_bleached(*λ*)_/*T*_coloured(*λ*)_)

*Q*_d_ is the amount of charge that is inserted into the thin film which can be determined by integrating the curve area of current density *versus* time.

## Results and discussion

### Structure and morphology

**Fig. 1:**
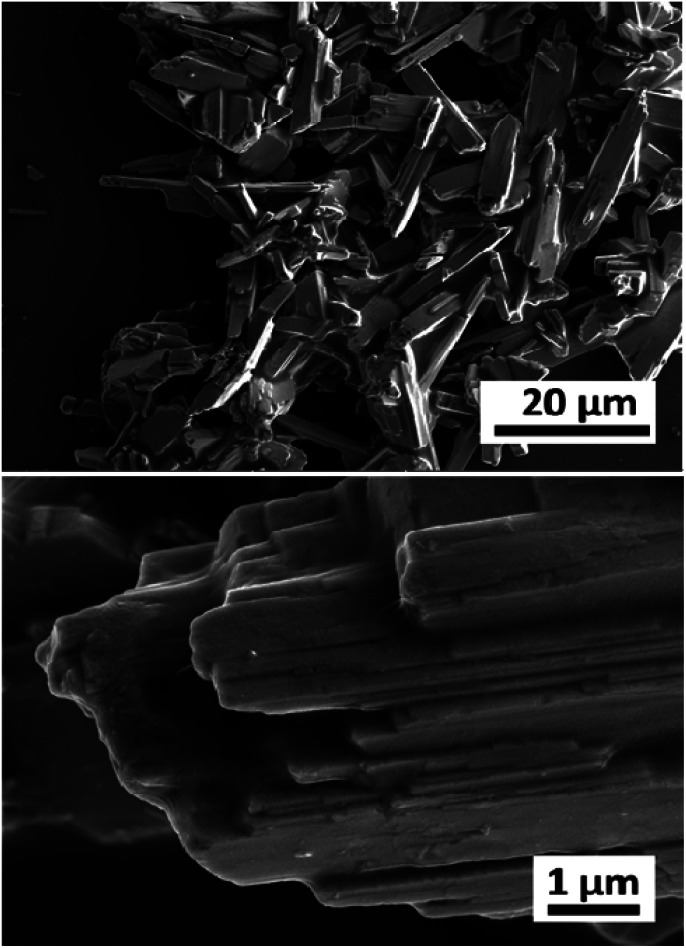
Low and high magnification SEM images of the pristine WO_*x*_ sample.

**Fig. 2:**
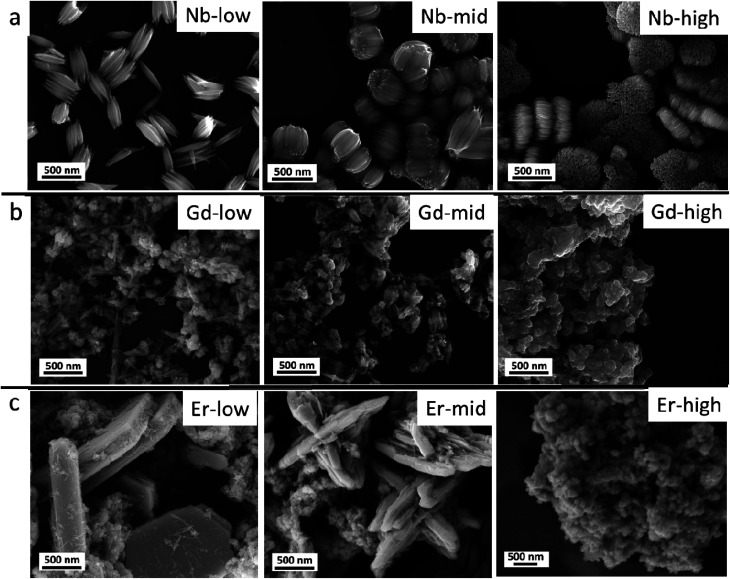
(a) Nb doped WO_*x*_, (b) Gd doped WO_*x*_ and (c) Er doped WO_*x*_ samples with low, mid and high concentration.

The pristine WO_*x*_ sample morphology (Fig. 1) presents needle-like units merging in a plate like with lengths of about 10 to 20 μm and widths of approximately 5–10 μm. The additional metal ions (Nb, Gd, and Er) significantly influence the morphologies as it can be seen from Fig. 2. All the doped samples present crystals in the nanometre range, whereas the ones in the pristine WO_*x*_ are characterized by a larger size in the micrometre range. As the concentration of Nb dopant (Fig. 2a) increase the nanowires became shorter (about 600 nm for Nb-low, 400 for Nb-mid and 100–200 nm for Nb-high). Additionally, these nanowires pack along the long axis to form tightly packed bundle-like structure with diameter of about 200, 400 and 600 nm for low, mid and high Nb concentration, respectively.

In the Gd doped WO_*x*_ samples (Fig. 2b), nanowires shorter (100–200 nm) than in the Nb doped series are detected at lower doping concentrations compared to those Nb doping samples. The sign of agglomeration is also observed in the Gd-low and Gd-mid sample, while in Gd-high the nanowires leave the space to nanoparticles.

In the Er series (Fig. 2c), a mixture of agglomerated nanowires and polygon-plated microstructures, with dimensions of 2 μm × 1 μm × 500 nm, are observed in the Er-low sample. Interestingly, in the Er-mid sample, a cross-shaped structure measuring approximately 2–3 μm in diameter and 1–2 μm in height can be observed. However, with Er-high, similarly to the Gd doped series, the morphology completely changes, transitioning into nanoparticle form.

**Fig. 3:**
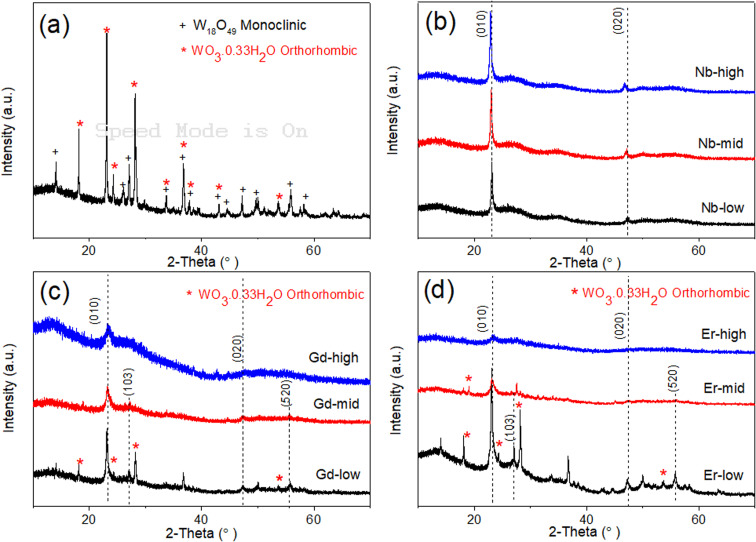
XRD patterns for WO_*x*_ based samples prepared *via* solvothermal method at 200 C, 10 h reaction. (a) Pure WO_*x*_ (b) Nb doped WO_*x*_ (c) Gd doped WO_*x*_ and (d) Er doped WO_*x*_ sample with different molar ratio.

The XRD pattern of pure WO_*x*_ (Fig. 3a) suggests that the as-prepared sample present two different phases of WO_*x*_: orthorhombic WO_3_0.33H_2_O (called o-WO_3_ from now on, crystallography open database reference COD 00-100-4050) and monoclinic W_18_O_49_ (called m-W_18_O_49_ from now on, COD 00-100-1678). The obtained peaks at 18.1°, 23.1°, 24.2°, 28.1°, and 53.7° correspond to planes (111), (002), (200), (220) and (204) of o-WO_3_, respectively, while the peaks at 14.0°, 26.1°, 27.1°, 33.7°, 44.5°, 47.2°, and 55.9° are attributed to planes (002), (503), (103), (404), (113), (903) and (520) of m-W_18_O_49_, respectively.

When looking at the doped-WO_*x*_ diffractograms, all the Nb doped WO_*x*_ samples show a pattern consistent with m-W_18_O_49_, with the most intense peaks located at around 23.1° and 47.5° (Fig. 3b). However, both peak locations exhibit a slight shift to lower 2-theta values when compared with the standard peaks at 23.4° and 48.0°. Interestingly, the 2-theta value decreases as the dopant amount increases. The shift observed in the XRD pattern is attributed to the lattice enlargement due to the Nb being slightly larger than W (0.64 *vs.* 0.60 Å in 6-fold coordination),^26–28^ resulting in a decrease in the diffraction angle with an increasing of the lattice constant. On the other hand, with the Gd-low and Er-low samples, mixed phases of m-W_18_O_49_ and o-WO_3_ are obtained, while XRD patterns similar to the Nb series were observable for the mid and high concentrations of Gd and Er (Fig. 3c and d). However, in the latter the peaks shift slightly to higher angles as the amount of dopant increases, as shown in Fig. S1a and b.† This trend, opposite to the Nb series, can be explained considering that the ionic radii of Er and Gd are much larger than the one of W (0.89 and 0.94 Å respectively),^29^ besides having lower oxidation states. Consequently, the substitution of tungsten with these two lanthanide elements, possible only at low concentrations, induces different, and more intense and distortions than Nb. The Nb-mid and Nb-high materials present the same crystallinity of Nb-low. Moreover, the diffractograms show a continuous shift of the (010) peak towards lower angle as the dopant concentration increase, suggesting that Nb effectively substitutes W in all the samples.

On the other hand, the mid and high concentrations of Er and Gd induce an amorphization of the oxide matrix, as indicated by the corresponding diffractograms, possibly due to the impossibility of accommodating high concentrations of large ions without destructing the inorganic WO_*x*_ frame.

**Fig. 4:**
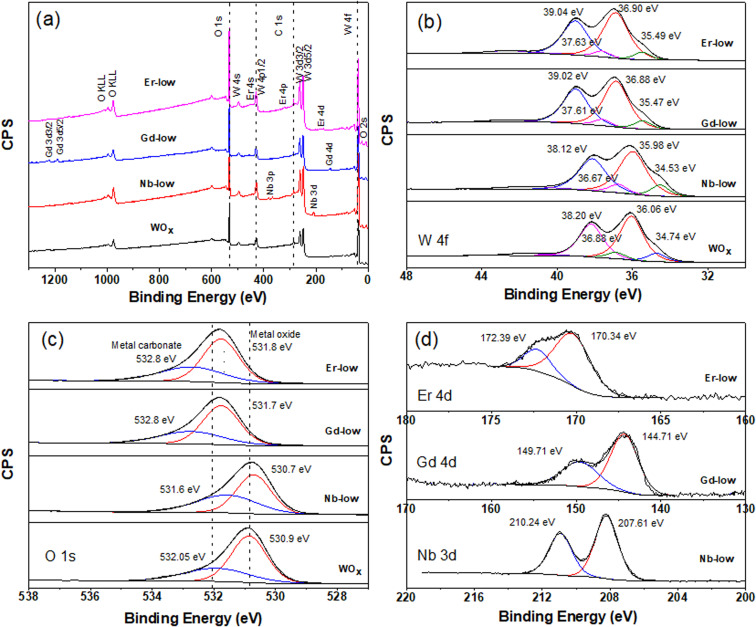
XPS spectra of all WO_*x*_ based nanomaterials with low concentration of dopant inside the structure, (a) survey XPS spectra (b) W 4f XPS spectra (c) O 1s XPS spectra and (d) Nb 3d, Er 4d and Gd 4d.

Further analysis of the surface chemical composition of the materials confirm that all metals have been successfully doped into the WO_*x*_ structures (Fig. 4a and S2–S4a†). The atomic percentages of elements from each sample are presented in Table 1. Moreover, we also observe a shift in the binding energy of the doped samples, which could be caused by the differences in oxidation state, electronegativity and ionic radii between the dopant (Nb, Gd, or Er) and the base element (W), leading to lattice strain and changes in the local bonding environment within the structure. The W 4f core-level spectra of all samples can be accurately fitted into two spin–orbit doublets, corresponding to two different oxidation states, namely W^5+^ and W^6+^ species (Fig. 4b and S1–S3b†). Two primary doublets at binding energies of approximately 36.06 eV for W 4f_7/2_ and 38.20 eV for W 4f_5/2_ are attributed to W^6+^ species. The other doublets at 34.74 eV for W 4f_7/2_ and 36.88 eV for W 4f_5/2_ are identified as W^5+^ species. The binding energy difference between these two doublets is set at 2.14 eV, maintaining the same full width at half maximum (FWHM) value, which is consistent with the literature.^30,31^

**Table 1:** Summary of atomic percentage of WO_*x*_-based materials from XPS^a^

Samples	Surface composition atomic ratio			
	W^5+^/W^6+^	O_total_ (M + W + O)	W_total_ (M + W + O)	M_total_ (M + W + O)
Pure WO_*x*_	0.136	75.90	24.01	

**Nb doped WO**_***x***_				
Low	0.146	72.98	25.99	1.03
Mid	0.124	74.73	23.17	2.10
High	0.164	75.89	20.37	3.74

**Gd doped WO**_***x***_				
Low	0.120	72.77	26.14	1.10
Mid	0.084	72.17	26.26	1.57
High	0.093	76.51	20.76	2.73

**Er doped WO**_***x***_				
Low	0.106	73.51	25.91	0.57
Mid	0.113	75.00	24.00	0.84
High	0.078	75.94	21.76	2.30

a M is the metal element inside the WO_*x*_ structure.

The formation of low valence states (W^5+^) is often accompanied by the emergence of oxygen vacancies (V_O_).^32^ The formation of V_O_ inside the structure is further confirmed by the O 1s core level which can deconvoluted into 2 main peaks (Fig. 4c and S2–S4c†). The peak is located at lower binding energy of about 530 eV attributing to lattice oxygen (O^2−^) in the metal oxide structure. The higher binding energy at about 532 eV is associated with O^2−^, OH^−^ in the oxygen deficient region of WO_*x*_ based samples. Hence, the appearance of O 1s from OH^−^ can indicate the presence of V_O_ in the structure.^32,33^ As shown in Table 1, a change in molar ratio of W^5+^/W^6+^ after introducing different doping elements are observed. This suggests that the dopants substitute the tungsten ions or form intercalated or intermixed structures within the lattice, causing lattice distortions and creating new localized electronic states within the WO_*x*_ structure.^34,35^ It is also worth mentioning that Pauling electronegativity of W (2.36) is higher than that of metal elements M (1.6, 1.2 and 1.24 for Nb, Gd and Er, respectively).^36^ Therefore, the difference in the electronegativity of the M–O bond is larger compared to W–O in the M–O–W bridge. Thus, the W–O bond is more polarizable compared to those metal–O bonds.

A shift in binding energy has been observed in samples doped with Nb, Gd, and Er compared to pure WO_*x*_ samples, confirming the electron exchange process between the metal dopants and surrounding atoms within the host structure (see Fig. 4, S2 and S3†). Looking at the Gd-low and Er-low samples it can be observed that the O 1s and W 4f peaks are shifted towards higher energy (red shift) compared to pure WO_*x*_. Furthermore, a similar and continuous trend is also observed with an increase in the amount of dopant within the host structure (Fig. S3 and S4†). The Gd 4d and Er 4d peaks exhibit a blue shift: the Gd 4d energy peak appears at a range of 144–144.5 eV and 149.2–149.7 eV for Gd 4d_5/2_ and Gd 4d_3/2_, respectively with various Gd dopant concentrations (Fig. 4d and S3d†). For Er 4d core level, two main peaks can be observed at about 170 and 172.25 eV corresponding to Er 4d_5/2_ and Er 4d_3/2_, respectively (Fig. 4d and S4d†). No trend between shift and dopant concentration are observed in the Gd and Er doped samples. Based on the analysis of the XPS spectra, we propose that the Er and Gd serve as charge acceptors from neighbouring atoms, increasing the amount of W^6+^. Interestingly, in the case of low concentrated Nb doped samples the opposite trend is observed: blue shift for O 1s and W 4f while red shift for Nb 3d compared with pure WO_*x*_ (Fig. 4). We suggest that Nb, in low concentration, acts as a donor, donating electron to the system. However, as the Nb concentration increases the behaviour is the opposite, resulting in red shift of all region (*i.e.* O 1s, W 4f, and Nb 3d, Fig. S2†). Possibly, Nb starts to form different types of defects or complexes (*e.g.* replacing oxygen) within the WO_*x*_ matrix with a consequent modification of the electron distribution, but more experimental and computational studies are required to validate this hypothesis. The Nb 3d core level spectra show Nb 3d_5/2_ and Nb 3d_3/2_ doubles peaks appearing at about 207 and 210 eV, respectively (Fig. 4d and S2d†) indicating as Nb^5+^ species with a spin–orbit splitting of 2.7 eV. The line shape remains constant in all different Nb concentrations, with the binding energy slightly increasing with the increasing of the Nb amount (*i.e.* 0.78 and 0.68 eV increase for lattice oxygen and oxygen deficient region, respectively).

### Electrochemical and optical properties

**Fig. 5:**
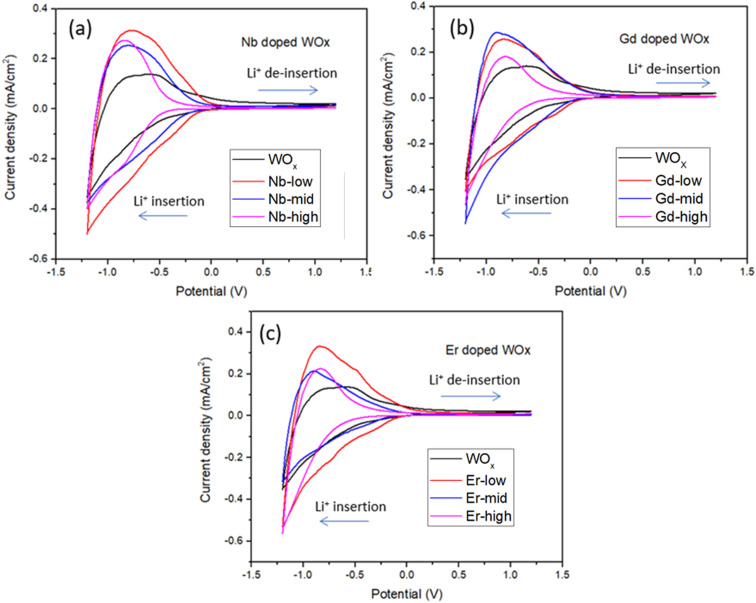
Cyclic voltammetry (2^nd^ cycles proceeding of Li^+^ insertion and de-insertion of film samples) for (a) Nb doped WO_*x*_ (b) Gd doped WO_*x*_ and (c) Er doped WO_*x*_ samples with low, mid and high molar concentration measured at the reversal points −1.2 to 1.2 V.

The film thickness is set at 500 nm ± 10 nm for all thin film samples. The spectroelectrochemical characteristics of the ECs are characterized using UV-Vis spectrophotometry under the application of 1.2 and −1.2 V bias with scan rate of 20 mV s^−1^. The charge capacity of all samples is presented as the cyclic voltammetry (CV) curve (Fig. 5). The best charge-capacity for insertion/de-insertion of different doping sample and pure WO_*x*_ are 0.49/0.32, 0.53/0.29, 0.53/0.33 and 0.54/0.22 mA cm^−2^ for Nb-low, Gd-mid, Er-low and WO_*x*_, respectively. The CV is also performed at different scan rate of 20, 40, 60, 80 and 100 mV s^−1^ at room temperature. The minimum and maximum value of current density from different scan rate of the CV are used and plotted against the square root of the scan rate *ν*^1/2^ (Fig. S5†). The slope from this graph was used to calculate the diffusion coefficient of Li^+^ (*D*_Li^+^_) based on eqn (1). The estimate of *D*_Li^+^_ for all samples are obtained in the range of 10^−10^ to 10^−12^ cm^2^ s^−1^ as presented in Table 2 and Fig. S6.† Additionally, summary of the tungsten oxide-based EC device from this study, in comparison to the literature also presented in ESI as shown in Table S1.†

**Table 2:** Summary of electrochromic properties for undoped and doped WO_*x*_ based films at 2^nd^ cycle of 20 mV s^−1^ CV scan rate

Samples	Diffusion coefficient (*D*_Li^+^_, 10^−10^, cm^2^ s^−1^)		Δ*T* (%), at 560 nm	Δ*T* (%), at 630 nm	Δ*T* (%), at 710 nm	Reversibility (%)	Responding time (s)		CE (cm^2^ C^−1^) from CV scan
	*D*_Li^+^ insertion_	*D*_Li^+^ de-insertion_					*t*_bleached_	*t*_coloured_	
WO_*x*_	0.47	0.38	7.2	13.8	20	89	1.2	12.9	34.1
Nb-low	2.60	2.49	29.6	42.7	54	99	1.0	8.4	49.3
Nb-mid	2.12	1.91	4.5	22.7	40	97.5	1.9	13.4	50.1
Nb-high	2.12	1.35	1.2	13.3	26	90	1.3	12.1	38.7
Gd-low	1.75	0.89	10.7	22.8	34	91	2.3	15.8	42.8
Gd-mid	1.82	1.56	8.2	21	32	97	1.1	12.7	41.6
Gd-high	0.074	0.043	0.9	5.3	10	91	1.1	16.5	23.5
Er-low	1.78	1.43	25.2	42.5	45	94	1.7	16.1	44.55
Er-mid	1.12	0.78	11.1	22.3	33	90	1.2	12.7	57.58
Er-high	0.035	0.023	0.9	3	8	92	1.3	18.9	16.3

The Li^+^ diffusion constant in the doped samples is higher than in the pure WO_*x*_ sample, exception made for Gd-high and Er-high. It is clear that the metal dopants can reduce the activation barrier for the Li^+^ ion insertion/de-insertion. It is worth mentioning that the improvement of ion diffusion kinetics is more evident with samples presenting good crystallinity and predominant o-WO_*x*_ structure. This is understandable observing the doped-low and -mid *D*_Li^+^_, all improved compared to pure WO_*x*_, but with Nb values higher than Er and Gd. On the other hand, when the samples become amorphous (Er-high, Gd-high) the diffusion constants drop, even compared to pure WO_*x*_. This behaviour can be appreciated observing the trend of the (010) peak intensity and diffusion constants with the dopant concentration in Fig. S7.† A possible explanation can be found through a slight enlargement of the WO_*x*_ matrix facilitating the Li^+^ ions movement, increasing the corresponding diffusion constants. On the other hand, when the distortion of the WO_*x*_ cage is too much, leading to amorphization, the movement of the Li^+^ ions is obstructed, with the consequent drop of diffusion values.^37^

The EC reversibility of the samples is estimated by using the ratio of charge de-insertion (*Q*_out_) to charge insertion (*Q*_in_) in the films. All the doped samples present higher reversibility than the pristine WO_*x*_ sample (10% increase in Nb-low), but higher metal ions amount result in a decrease of the performances. The clear similarity between the behaviour of reversibility and diffusion constant indicates that the crystallographic changes play a similar role in the two parameters. It is important to note, however, that the variability of the reversibility values at mid and high dopants concentration indicates that other additional factors, such as polarization events, dead-zone formation *etc.*, can sensibly affect this parameter.^38^

**Fig. 6:**
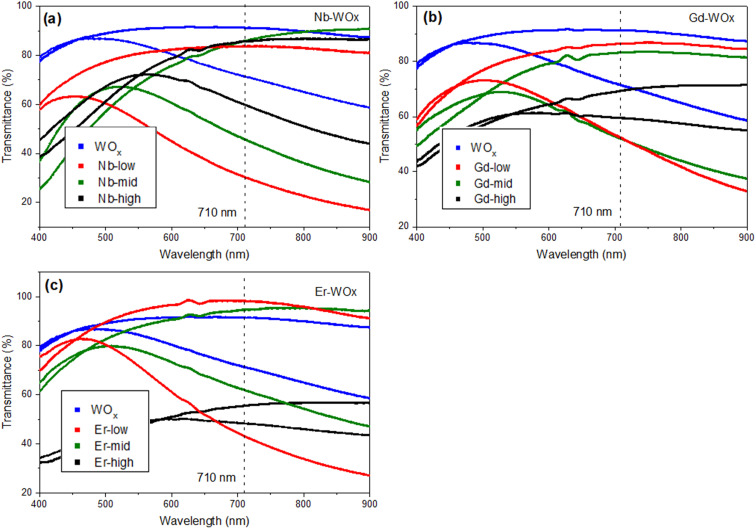
Transmittance spectra of (a) Nb doped WO_*x*_ (b) Gd doped WO_*x*_ and (c) Er doped WO_*x*_ thin films measured in contact to LiClO_4_ in PC at a 2^nd^ cycle of CV with scan rate of 20 mV s^−1^, in the bleached (1.2 V) and in the coloured (−1.2 V) states.

The transmittance change percentage (Δ*T*%) at a given specific wavelength of 560, 630 and 710 nm can be calculated using eqn (2).^25,39,40^ As observed in Fig. 6 and Table 2, the Δ*T*% values increase with wavelength. The Δ*T*% values follow a trend similar to *D*_Li^+^_: all the doped samples show higher Δ*T*% than the pure WO_*x*_ sample with the exception of Gd- and Er-high. The small change in the optical transmittance and poor electrochemical performance of undoped WO_*x*_ and dopant-high samples (Δ*T*% of Nb-high is only slightly higher than the WO_*x*_ pure), could be due to the strong Coulomb ion lattice interaction,^41^ leading to the ineffective insertion of Li^+^ into WO_*x*_. Moreover, it could be possible that Nb, Gd and Er ions in high concentrations block the access to the active layer, as the multivalent ions have a larger size than monovalent Li^+^.^42^

**Fig. 7:**
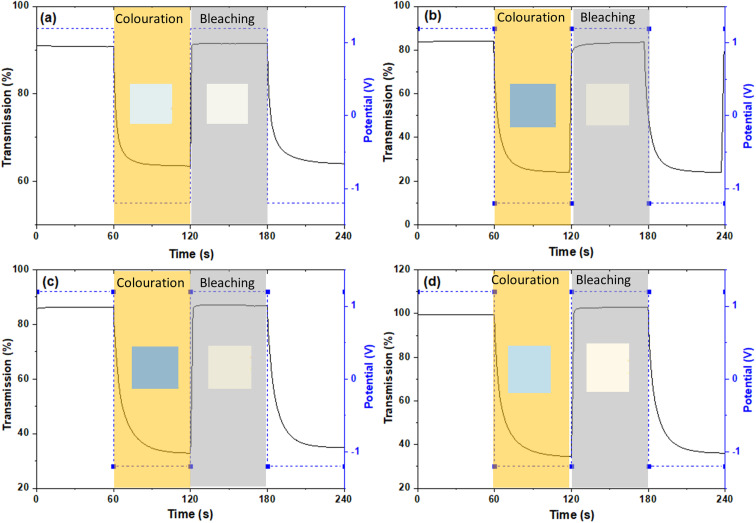
*In situ* optical transmittance of (a) pure WO_*x*_ (b) Nb doped WO_*x*_ (c) Gd doped WO_*x*_ and (d) Er doped WO_*x*_ with lowest concentration (1 : 16) at 710 nm in potential steps of −1.2 to 1.2 V. The inset-coloured squares represent the coloured and bleached stages, as defined by the Red Green Blue (RGB) colour system.

The colouration efficiency (CE) is extracted as the slope of the line fitting the linear region of the curve of the optical density of (ΔOD) based on eqn (3). The CE is a crucial parameter in the development of EC devices as it indicates the EC device's optical modulation capability with respect to the intercalation charge density. In practical applications, a high CE value is desirable as it enhances the long-term stability of EC devices by reducing the required charge insertion or extraction.^43^ As expected by the previously discussed Δ*T*%, the sample with low and mid amount of dopant show better CE value compared with undoped and highly doped samples, independently from the element type. The transmission percentage *vs.* time of all samples are presented in Fig. 7 and S8.† The switching times of the film between the coloured (*T*_coloured_) and bleached (*T*_bleached_) states were evaluated using the time in which 90% change in transmittance modulation at 530, 630 and 710 nm is achieved and the results are also summarised in Table 2 and Fig. S9.† The bleaching process is faster than the colouration process in all cases. Moreover, the samples presenting fast colouring also bleach quickly. However, a clear trend between the differences in transmittance (Δ*T*%) values or switching time and the concentration of dopants was not observed. This absence of a trend may indicate the presence of more complex phenomena, potentially related to hysteresis.^44^ The lowest concentration of Nb doped WO_*x*_ offer the best switching time which values of 8.4 and 1.0 s for coloured and bleached states, respectively, compared with the other samples as shown in Fig. S8 and S10.† We suggest that, beside the dopant-induced crystallographic changes previously discussed, the morphology could also play a crucial role in the colour switch behaviour. The Nb-low sample offers a higher surface area compared to the best EC performance samples from other dopant categories, namely Gd-mid and Er-low (see Fig. 2). It's worth noting that the Gd-mid also exhibits an agglomerated structure, which further reduces the surface area, essential to facilitate a shorter path for Li^+^ ions to penetrate the WO_*x*_ structure. Moreover, it is interesting to note that, despite the lower *D*_Li^+^_ for the de-insertion than the insertion process, the bleaching switching time (*i.e.* the de-insertion process) is faster than the colouring switching time (*i.e.* the insertion process). The presence of residual Li^+^ ions could be a factor affecting this behaviour, but more evidences are needed to explain these phenomena.

## Conclusion

Doping is an efficient way to enhance the performance of metal oxide-based EC materials, as demonstrated in our investigation of Nb, Gd, and Er doping on WO_*x*_ structure. Our findings suggest that the amount and size of metal dopant are a key factor in maximizing the performance. Our structural investigations confirm that low-doping concentrations result in a distorted structure compared to the pure WO_*x*_, which facilitate the Li^+^ insertion/de-insertion with consequent better EC performance. Our results show significant improvements in the insertion/de-insertion current density and coloration efficiency of WO_*x*_-based EC materials with metal doping, specifically an improvement of about 5, 4 and 3.5 times for Nb, Gd, and Er doping samples, respectively compared to pure WO_*x*_, in terms of intercalation/de-intercalation current density, and improvements of 16, 9, and 24% for coloration efficiency of Nb, Gd, and Er doping samples, respectively compared to pure WO_*x*_. Our results show that a slight distortion of the matrix, due to a low amount of dopant is preferable than higher amounts. When higher concentrations are used, the size of the dopants play an important role, with dopants having similar size to W performing better than bigger ones. Further research is necessary to address the remaining challenges associated with EC materials, but our study provides promising insights towards the development of more efficient EC technology for energy-efficient buildings. Our investigation highlights the potential of metal doping on WO_*x*_ for optimizing insertion/de-insertion current density and coloration efficiency in EC materials. Future research can build upon these findings to continue improving the performance of EC glass, ultimately contributing to the development of more sustainable and energy-efficient buildings.

## Author contributions

KT was responsible for the project ideas including the experiments, analysis of the result and preparing the manuscript. TN and DS contributed on the EC performance measurements and interpretation of the data which was collect from their laboratory. GL, GZ, YQ and NW were responsible for reviewing and revising the manuscript. DQ supplied the XPS data. NF supported on XPS data analysis. PM supported on XRD and SEM measurements. All authors have reviewed and given approval to the final version of the manuscript.

## Conflicts of interest

There are no conflicts to declare.

## Supplementary Material

RA-013-D3RA06018G-s001
