# Supplementary material for: Effect of metal dopants on the electrochromic performance of hydrothermally-prepared tungsten oxide materials
Source: RSC Adv. 2023 Dec 19;13(50):35457–67. doi: 10.1039/d3ra06018g (PMC10728781; doi:10.1039/d3ra06018g)
Supplement: RA-013-D3RA06018G-s001 [file RA-013-D3RA06018G-s001.pdf]

Electronic Supplementary Material (ESI) for RSC Advances.
This journal is © The Royal Society of Chemistry 2023

Supporting information

Effect of metal dopants on the electrochromic performance of hydrothermally-prepared tungsten oxide materials

Kunyapat Thummavichai*[,1], Thi Hai Quyen Nguyen[2], Giulia Longo[1], Dayuan Qiang[3], Guillaume Zoppi[1] and Derck Schlettwein[2], Pietro Maiello[1], Nicole Fleck,[1] Nannan wang[4], Yanqiu Zhu[5]

1 Department of Mathematics, Physics and Electrical Engineering, Faculty of Engineering and Environment, Northumbria University, NE1 8ST, Newcastle, UK

2 Institute of Applied Physics and Center for Materials Research (ZfM/LaMa), Justus-Liebig University Giessen, Heinrich-Buff-Ring 16, Giessen, Germany

3 Department of Mechanical Engineering Sciences, University of Surrey, GU2 7XH, Surrey, UK

4 State Key Laboratory of Featured Metal Materials and Life-cycle Safety for Composite Structures, School of Resources, Environment and Materials, Guangxi University, Nanning, 530004, China.

5. College of Engineering, Mathematics and Physical Sciences, University of Exeter, Exeter EX4 4QF, UK

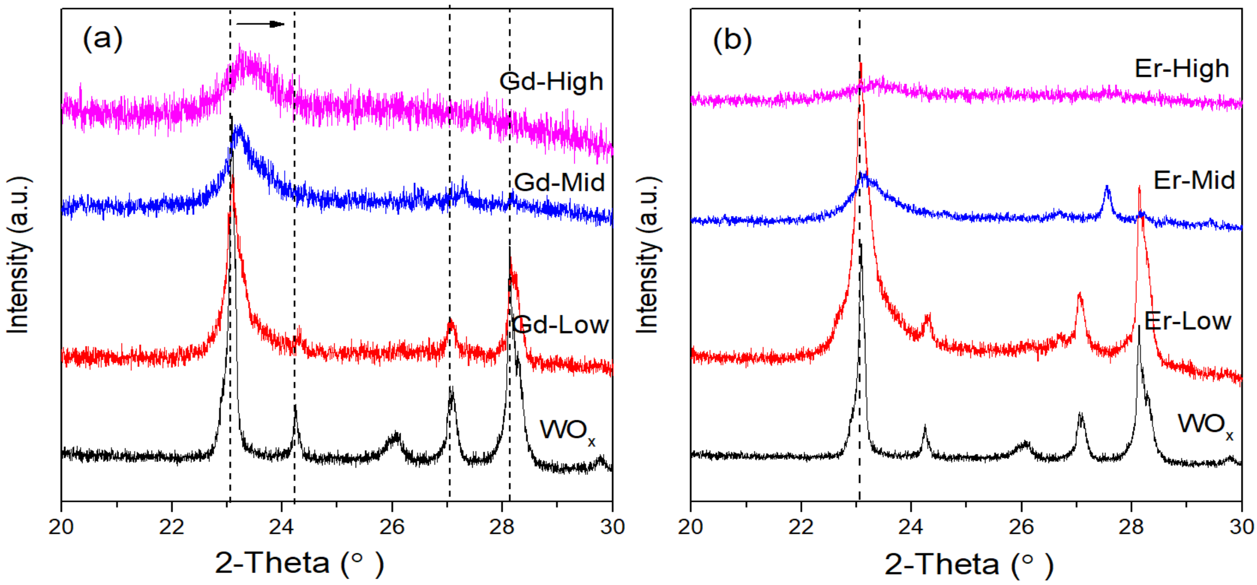

Fig S1. XRD spectra of (a) Gd and (b) Er doped WO_x samples with high, mid and low additional metal concentration.

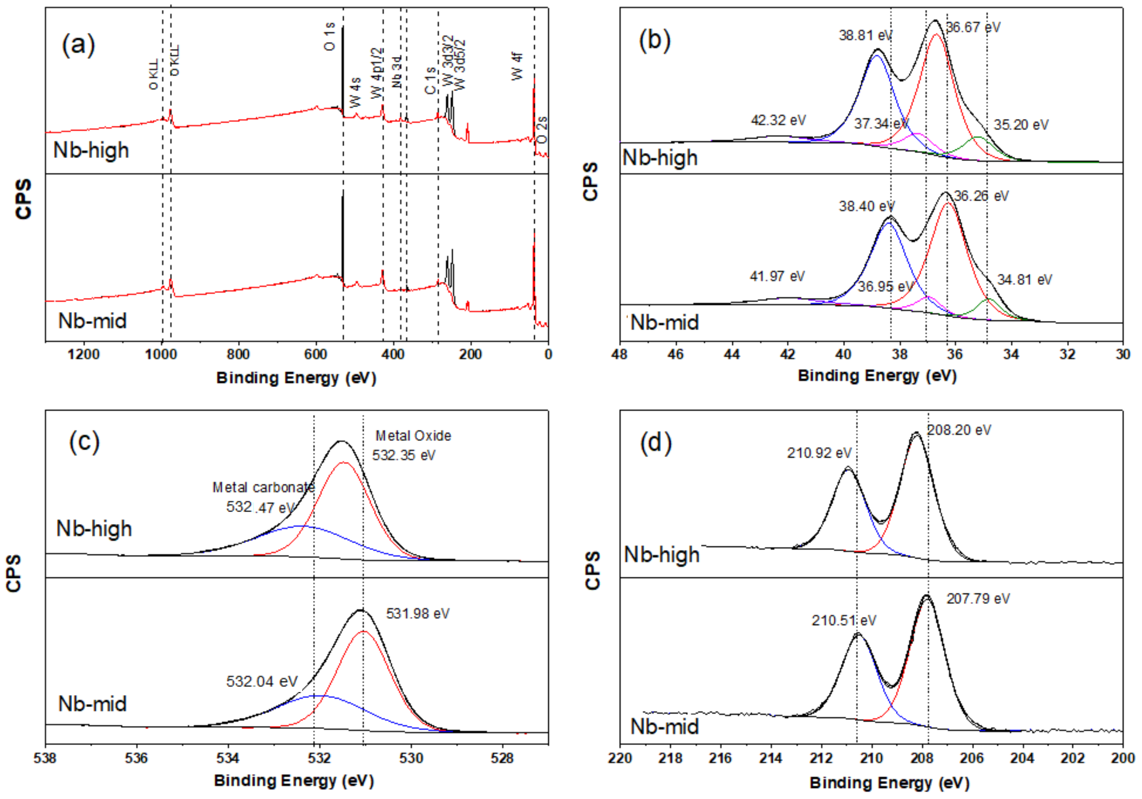

Fig S2. XPS spectra of Nb doped samples with mid and high concentration molar ratio (a) survey (b) W 4f, (c) O 1s and (d) Nb 3d spectra.

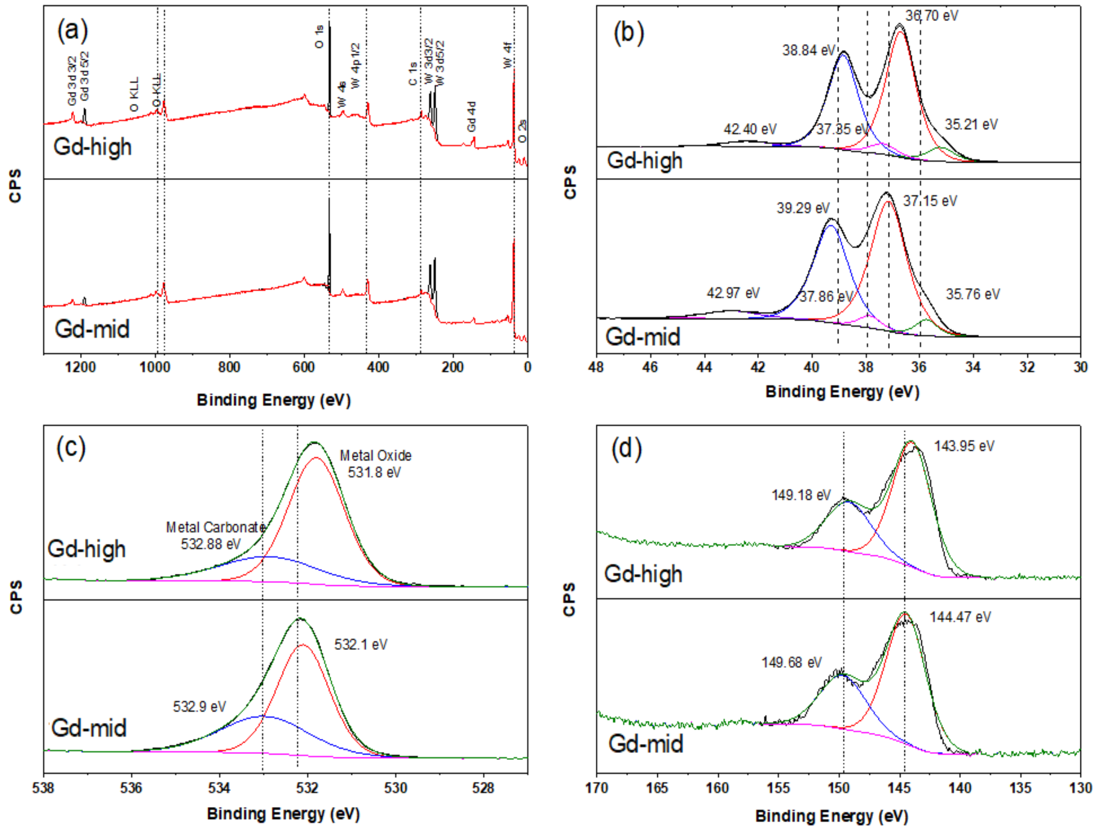

Fig S3. XPS spectra of Gd doping samples with mid and high concentration molar ratio(a) survey (b) W 4f, (c) O 1s and (d) Gd 4d spectra.

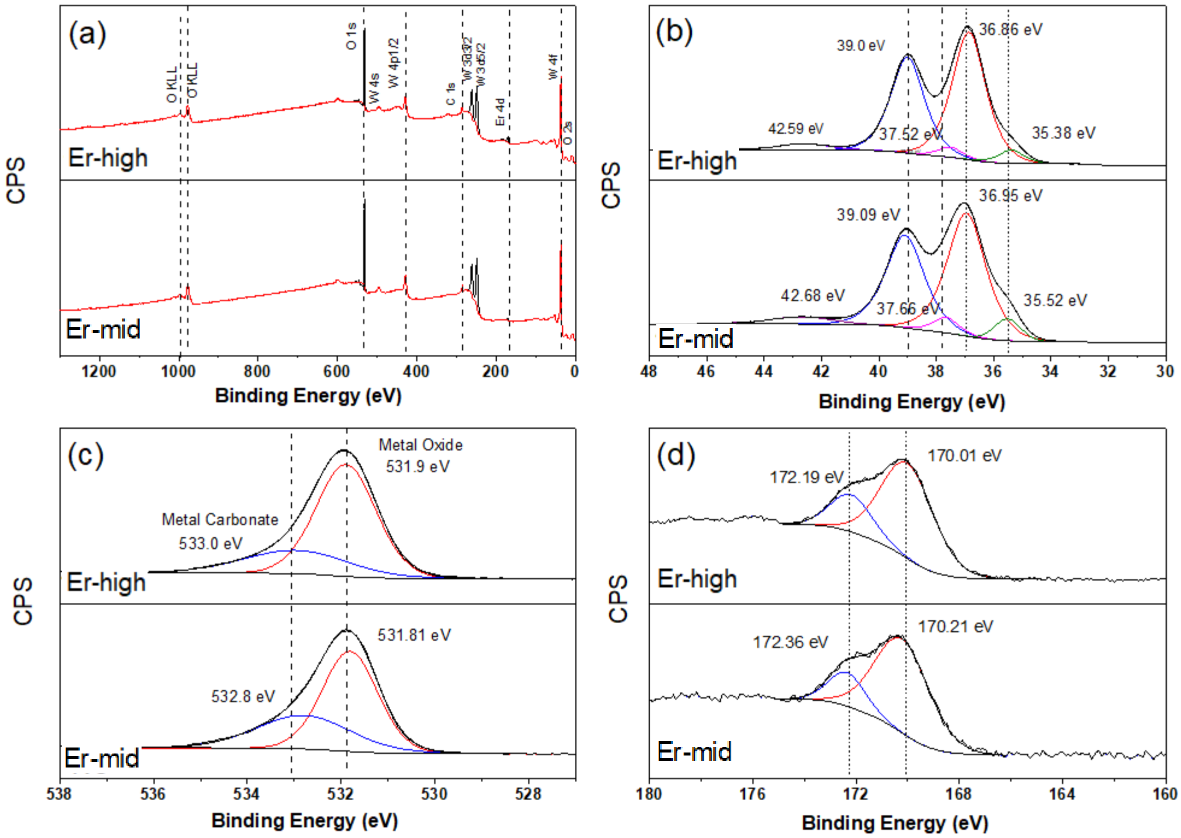

Fig S4. XPS spectra of Er doping samples with mid and high concentration molar ratio (a) survey (b) W4f, (c) O 1s and (d) Er 4d spectra

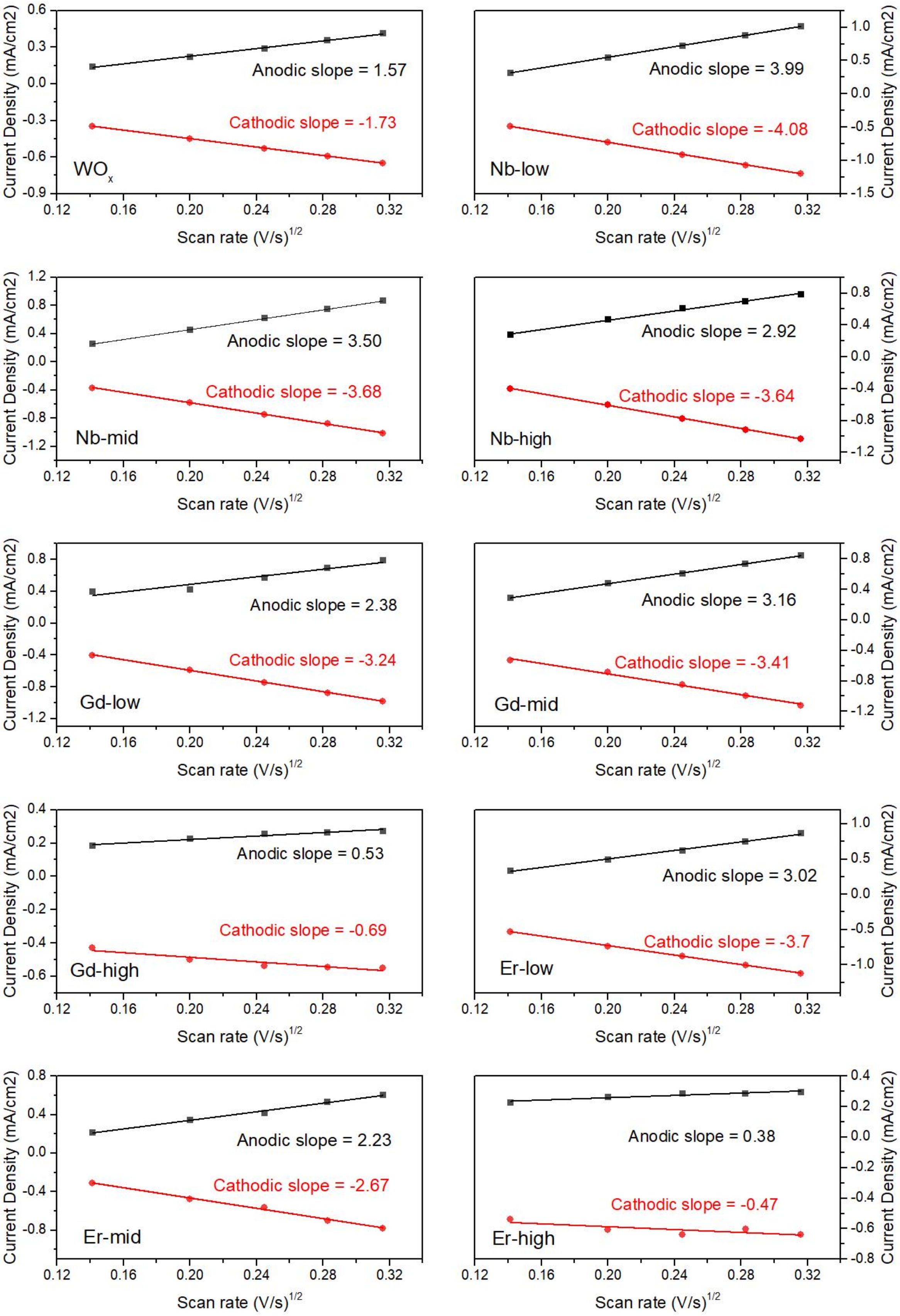

Fig S5. Current density from different scan rate of the CV plotted against the square root of the scan rate $v^{1/2}$ for all samples.

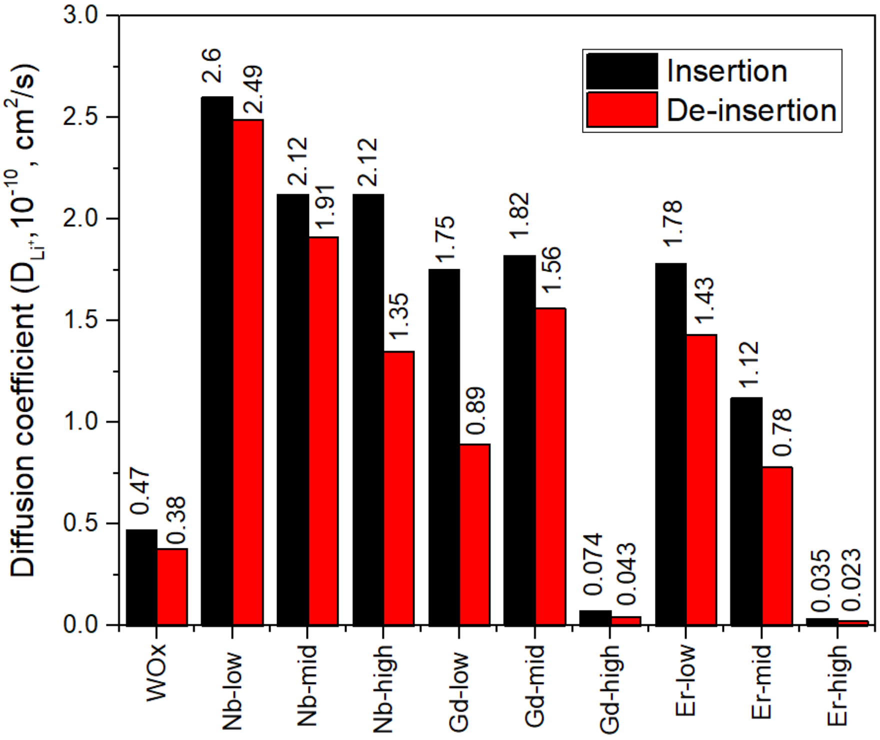

Fig S6. Ion diffusion constant of all samples.

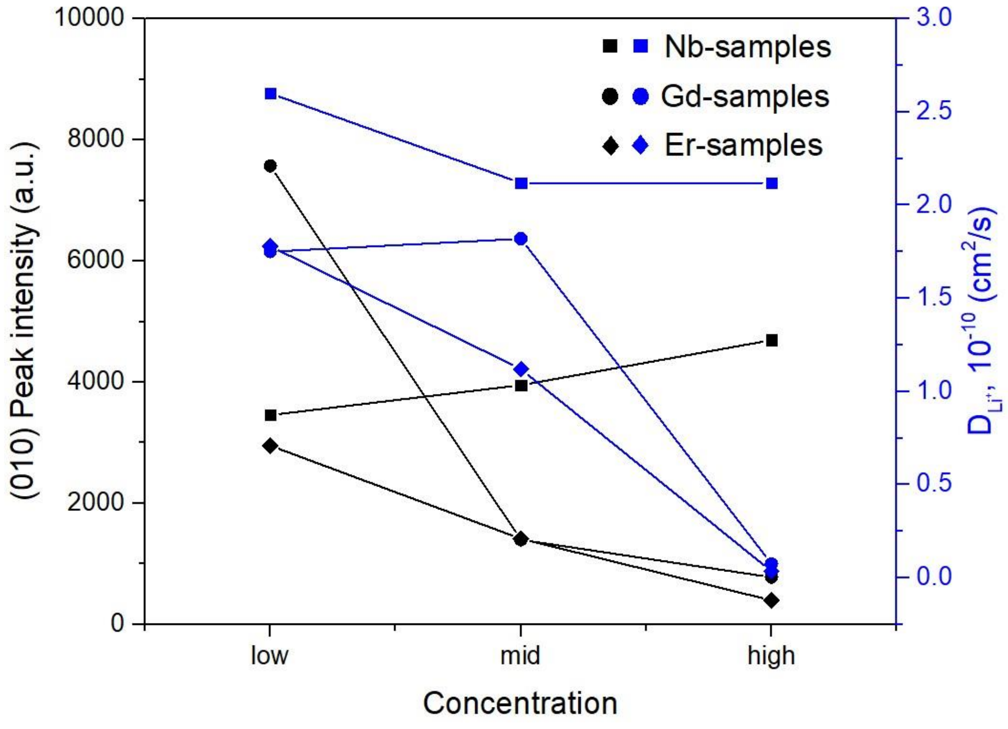

Fig S7. Ion diffusion constants of the insertion process relative to structural characteristics across all samples.

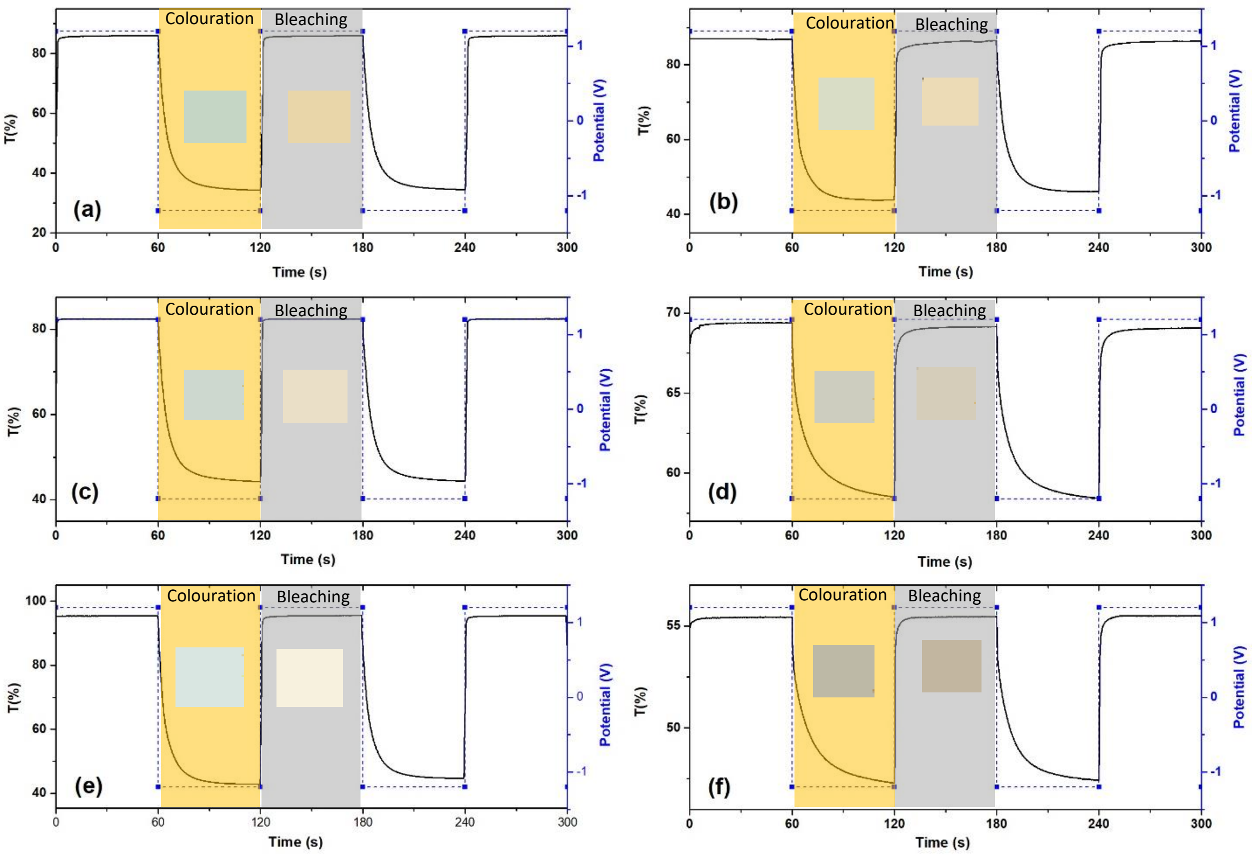

Fig S8. Optical transmittance at 710 nm of (a) Nb-mid, (b) Nb-high, (c) Gd-mid, (d) Gd-high, (e) Er-mid and (f) Er-high, recording via chronoamperometry. The inset-coloured squares represent the coloured and bleached stages, as defined by the Red Green Blue (RGB) colour system.

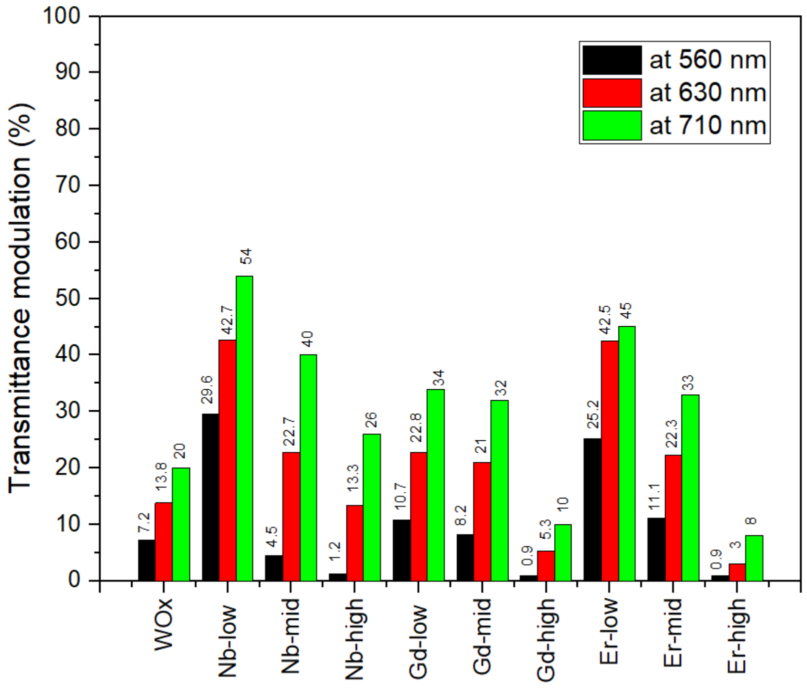

Fig S9. Transmittance modulation of all samples at 560, 630 and 710 nm.

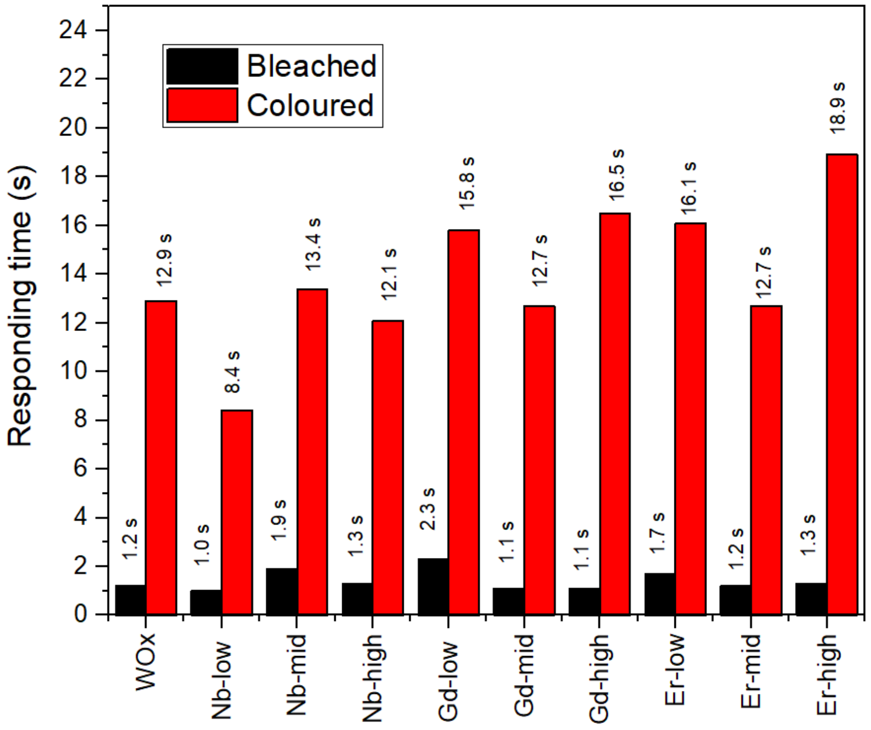

Fig S10. Responding time of all samples.

Table S1: Summary of the tungsten oxide-based EC device from this study, in comparison to the literatures.

**Samples**	**Reversibility (%)**	**Colour efficiency (cm^2/C)**	**Responding time**		**Durability (cycles)**	**References**
			Coloured	Bleached		
**WO_x**	89	34.1	12.9	1.2	-	This work
**Nb doped WO_x (1:16)**	99	49.3	1.0	8.4	-	This work
**Gd doped WO_x (1:16)**	91	42.8	2.3	15.8	-	This work
**Er doped WO_x (1:16)**	94	44.55	1.7	16.1	-	This work
**WO_3**	-	60.3	4.3	11.6	1000	[1]
**WO_3**	75	25	3	5	-	[2]
**$Nb_{18}W_{16}O_{93}$**	-	46.57	4.7	4.0	8000	[3]
**6%Nb-WO_3**	92	7	4.3	5.6	-	[2]
**5%Nb-WO_3**	-	76.2	4.1	10.8	1000	[1]
**2%Gd-WO_3**	-	43	11.2	18.5	-	[4]
**Gd-WO_3 (0.29at%)**	-	71.4	1.6	3.2	2000	[5]

References

1. W. Q. Wang, Z. J. Yao, X. L. Wang, X. H. Xia, C. D. Gu and J. P. Tu, *Journal of Colloid and Interface Science* **2019**, *535*, 300-307.
2. S. R. Bathe and P. S. Patil, Journal of *Applied Physic D* **2007,** *40*, 7423.
3. C. Wu, Z. Shao, W. Zhai, X. Zhang, C. Zhang, C. Zhu, Y. Yu and W. Liu, *ACS Nano* **2022,** *16*, 2621-2628.
4. Y. Yin, C. Lan, S. Hu and C. Li, *Journal of Alloys and Compounds* **2018,** *739*, 623-631.
5. Y. Yin, T. Gao, Q. Xu, G. Cao, Q. Chen, H. Zhu, C. Lan and C. Li, *Journal of Materials Chemistry A* **2020,** *8* (21), 10973-10982.
